# Amrubicin Monotherapy for Patients with Platinum-Refractory Gastroenteropancreatic Neuroendocrine Carcinoma

**DOI:** 10.1155/2015/425876

**Published:** 2015-06-23

**Authors:** Takayuki Ando, Ayumu Hosokawa, Hiroki Yoshita, Akira Ueda, Shinya Kajiura, Hiroshi Mihara, Sohachi Nanjo, Haruka Fujinami, Jun Nishikawa, Kohei Ogawa, Takahiko Nakajima, Johji Imura, Toshiro Sugiyama

**Affiliations:** ^1^Department of Gastroenterology and Hematology, Faculty of Medicine, University of Toyama, Sugitani, Toyama 2630, Japan; ^2^Department of Diagnostic Pathology, Graduate School of Medicine and Pharmaceutical Sciences, University of Toyama, Sugitani, Toyama 2630, Japan

## Abstract

*Objective*. Patients with gastroenteropancreatic neuroendocrine carcinoma (NEC) have a poor prognosis. Platinum-based combination chemotherapy is commonly used as first-line treatment; however, the role of salvage chemotherapy remains unknown. This study aimed to analyze the efficacy and safety of amrubicin monotherapy in patients with platinum-refractory gastroenteropancreatic NEC. *Methods*. Among 22 patients with advanced gastroenteropancreatic NEC, 10 received amrubicin monotherapy between September 2007 and May 2014 after failure of platinum-based chemotherapy. The efficacy and toxicity of the treatment were analyzed retrospectively. *Results*. Eight males and two females (median age, 67 years (range, 52–78)) received platinum-based chemotherapy, including cisplatin plus irinotecan (*n* = 7, 70%), cisplatin plus etoposide (*n* = 2, 20%), and carboplatin plus etoposide (*n* = 1, 10%) before amrubicin therapy. Median progression-free survival and overall survival after amrubicin therapy were 2.6 and 5.0 months, respectively. Two patients had partial response (20% response rate), and their PFS were 6.2 months and 6.3 months, respectively. Furthermore, NEC with response for amrubicin had characteristics with a high Ki-67 index and receipt of prior chemotherapy with cisplatin and irinotecan. Grade 3-4 neutropenia and anemia were observed in four and five patients, respectively. *Conclusion*. Amrubicin monotherapy appears to be potentially active and well-tolerated for platinum-refractory gastroenteropancreatic NEC.

## 1. Introduction

Gastroenteropancreatic neuroendocrine neoplasms (NENs) are currently classified into neuroendocrine tumors (NETs) and neuroendocrine carcinoma (NEC) on the basis of the morphology and proliferation rate. According to the 2010 WHO classification, NEC is defined as tumors with poorly differentiated morphology and a high proliferation rate, and it includes small cell type, large cell type, and small and large cell types [1]. The gastroenteropancreatic tract is the most common site for extrapulmonary NEC, accounting for 35% to 55% of all NECs outside the lung, although they are very rare [2, 3].

In general, chemotherapy is the main therapeutic strategy for gastroenteropancreatic NEC, and chemotherapy regimens developed for small cell lung cancer (SCLC) are recommended, because the clinical behavior of gastroenteropancreatic NEC is similar to that of SCLC. Combination chemotherapies of cisplatin plus etoposide or cisplatin plus irinotecan have been widely used for gastroenteropancreatic NEC on the basis of retrospective or small phase II studies [4–6]. In a recent retrospective study, 252 patients with advanced gastroenteropancreatic NEC received either cisplatin plus etoposide or carboplatin plus etoposide as a first-line treatment [7]. In this study, the response rate (RR) was 31%, the median progression-free survival (PFS) was 4 months, and the median overall survival (OS) was 11 months. No differences in treatment outcome were observed when comparing cisplatin-based chemotherapy with carboplatin-based chemotherapy. Furthermore, a Ki-67 index of >55% was reported to be predictive factors of response for platinum-based chemotherapy.

After first-line treatment, no further standard chemotherapy has been established in gastroenteropancreatic NEC. Retreatment with cisplatin plus etoposide has been reported to be a valid option after a treatment-free interval of at least 3 months [7]. Temozolomide with or without capecitabine and bevacizumab has also recently been reported to be effective in a cohort of 25 patients with NEC, especially in NEC with a Ki-67 index of <60%, after progression on cisplatin-based chemotherapy [8]. Amrubicin, a totally synthetic 9-amino-anthracycline that acts as a potent topoisomerase II inhibitor, has been developed for the treatment of SCLC. In the second-line setting, amrubicin monotherapy showed a RR of 17%–52% in patients with SCLC [9–11]. In five patients with gastrointestinal NEC, amrubicin monotherapy was shown to be potentially effective [12]. However, its activity for platinum-refractory gastroenteropancreatic NEC has not yet been described.

In this study, patients with advanced gastroenteropancreatic NEC were treated with platinum-based chemotherapy in the first-line setting and with amrubicin monotherapy in the salvage setting. Thus, we conducted a retrospective review of data from patients with gastroenteropancreatic NEC who had received second- or third-line amrubicin therapy to evaluate the efficacy and toxicity of this agent. Furthermore, we analyzed the clinicopathological characteristics of NEC in response to amrubicin therapy with regard to the Ki-67 index and previous chemotherapy.

## 2. Materials and Methods

### 2.1. Patient Selection

Patients with advanced gastroenteropancreatic NEC were retrospectively selected at our institutions between September 2006 and May 2014. All patients fulfilled the following criteria: (1) a histologically confirmed diagnosis of advanced gastroenteropancreatic NEC; (2) receipt of one or two prior treatments with platinum-based chemotherapy; (3) receipt of single-agent treatment with amrubicin as salvage chemotherapy.

### 2.2. Pathological Diagnosis

Pathologists at our institute reviewed all resected or biopsy samples for the study. The pathological diagnosis of gastroenteropancreatic NEC was established according to the histopathological criteria with a Ki-67 index of >20%, according to the WHO classification. Immunohistochemical analysis using a panel of neuroendocrine markers, including chromogranin A, synaptophysin, and CD56 (neural cell adhesion molecule), was performed before the first-line chemotherapy for all patients to confirm the neuroendocrine differentiation of the cancer cells.

### 2.3. Data Collection and Statistical Analysis

The patients' baseline characteristics, including age, gender, performance status, and data on the clinical stage of the disease, history of prior chemotherapy with or without radiation, dose of amrubicin, number of cycles, tumor response, toxicity, date of recurrence, and date of the last follow-up, were retrospectively obtained from medical charts. The clinical stage was reassessed according to International Union for Cancer Control (UICC) staging criteria, and tumor response was assessed on computed tomography (CT) every 2 months according to Response Evaluation Criteria in Solid Tumor guideline, version 1.1. Toxicity was evaluated according to the Common Terminology Criteria for Adverse Events (CTCAE v4.0). PFS was measured from the day of the initiation of amrubicin therapy to the day on which disease progression was confirmed or the final day of follow-up without disease progression. OS was measured from the day of the initiation of amrubicin therapy until the day of death or the final day of follow-up. PFS and OS rates were estimated by the Kaplan-Meier method. All statistical analyses were performed with the use of JMP version 10 (SAS Institute, Cary, NC).

This study was conducted with the approval of the Institutional Review Board of Toyama University, Toyama, Japan.

## 3. Results

### 3.1. Patient Characteristics

A total of 22 patients with advanced and recurrent gastroenteropancreatic NEC received first-line chemotherapy between September 2006 and May 2014. Of these, 21 patients received platinum-based chemotherapy according to the treatment strategy for SCLC and 10 patients received amrubicin as salvage chemotherapy. Specimens used for histological examination included surgically resected specimens in one patient and endoscopic biopsy specimens in nine patients. Among 11 patients that were not treated with amrubicin, eight patients had not received salvage chemotherapy, and three had received etoposide and carboplatin as second-line chemotherapy, respectively.

Patient characteristics immediately before the initiation of amrubicin therapy are summarized (Table 1). Surgical resection was performed only in one patient with esophageal NEC, and this patient developed lymph node metastasis 2 months after surgery. Five patients had received one previous course of chemotherapy, and the remaining patients had received two previous chemotherapy courses. All patients had received platinum-based chemotherapy as a first-line treatment, and a combination of cisplatin and irinotecan was the most frequently administered regimen prior to amrubicin therapy. Two patients with esophageal NEC received radiotherapy in addition to chemotherapy.

### 3.2. Treatment Results and Survival

**Figure 1:**
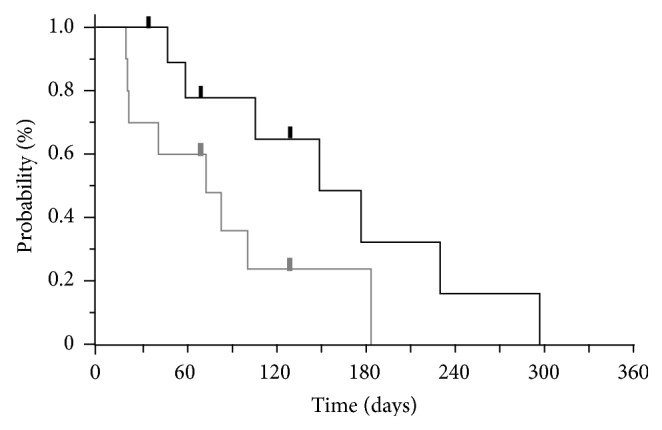
The median PFS and OS of study individuals.

The dose of amrubicin was determined at each physician's discretion, and six patients received 40 mg/m^2^/day for 3 days every 3 weeks and four patients received 35 mg/m^2^/day. A total of 30 cycles of amrubicin were administered to all patients, and the median number of cycles per patient was three (range, 1–7). Because of progressive disease, four patients required discontinuation of chemotherapy after only one cycle. As a result, the median PFS in all patients was 2.6 months (95% CI, 0.7–6.2) and the median OS was 5.0 months (95% CI, 1.5–9.9) after the initiation of amrubicin therapy (Figure 1).

### 3.3. Tumor Response

**Figure 2:**
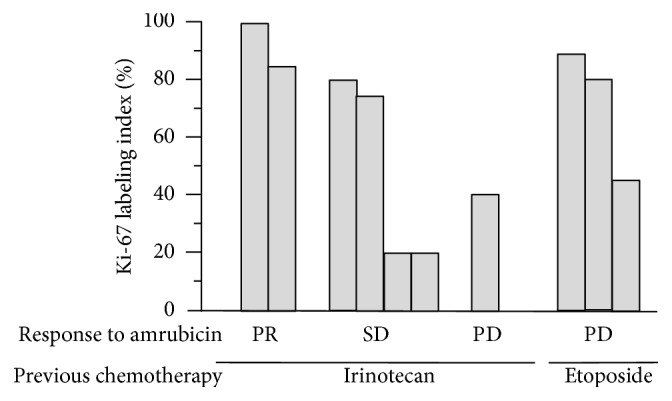
The association between response to amrubicin therapy and the Ki-67 index or previous chemotherapy.

The overall RR was 20% (95% CI, 4.7%–44.7%), and the disease control rate was 60.0% (95% CI, 29.6%–90.3%). Two patients with pancreatic or gastric NEC had a partial response, and their PFS was 6.2 months and 6.3 months, respectively. The tumor response, Ki-67 index, and first-line chemotherapy were illustrated to analyze the characteristics of NEC in response to amrubicin (Figure 2). Two patients with partial response had characteristics of NEC with a high Ki-67 index (99% and 89%, resp.), and they had received cisplatin and irinotecan as a first-line treatment. On the other hand, all three patients receiving cisplatin and etoposide as first-line treatment, including the topoisomerase II inhibitor, had progressive disease.

### 3.4. Toxicity

The most common adverse events were hematological toxicities; grade 3 or 4 leukopenia, neutropenia, and anemia were observed in two (20%), four (40%), and five (50%) patients, respectively (Table 2). Febrile neutropenia was not observed, and G-CSF was not used in any patients. Nonhematological toxicities were less frequent; grade 3 or 4 anorexia, nausea, and fatigue were observed only in one (10%) patient. No evidence of cardiac toxicity of amrubicin was observed, and no treatment-related deaths occurred.

## 4. Discussion

This retrospective study analyzed the efficacy and safety of amrubicin monotherapy in patients with platinum-refractory gastroenteropancreatic NEC. The present study showed a RR of 20.0% and a median PFS of 2.6 months. These outcomes were comparable with those reported for amrubicin chemotherapy for SCLC in the second-line setting; the reported ranges of RR and median PFS in phase II studies were 17–52% and 2.6–4.0 months, respectively [9–11, 13]. Considering these facts, amrubicin therapy appears to be potentially active for platinum-refractory gastroenteropancreatic NEC.

In this study, two patients showed partial response that led to long survival. These patients had NEC with a high Ki-67 index and received irinotecan plus cisplatin. Preclinical studies have suggested that treatment with topoisomerase I inhibitors results in downregulation of the topoisomerase I target and reciprocal upregulation of topoisomerase II, thereby leading to a response by topoisomerase II inhibitors [14–16]. Conversely, treatment with topoisomerase II inhibitors results in downregulation of topoisomerase II and upregulation of topoisomerase I. Among patients with SCLC, prior chemotherapy with etoposide was reported to be associated with a poor response and survival in a phase II study with amrubicin as a second-line treatment [13]. This may explain why prior treatment with irinotecan was associated with a response to amrubicin therapy in the present study.

According to the WHO classification, all patients in this study had a Ki-67 index of >20%. Although its classification does not distinguish between different levels of Ki-67 in NEC, there appears to be a difference in tumor biology between NEC with low and high Ki-67 indices [17]. Indeed, a Ki-67 index threshold of 55% was reported to be predictive for the response to first-line platinum-based chemotherapy [7]. Tumors with a Ki-67 index of <55% were much less responsive (RR, 15% versus 42%), but the patients with these tumors had a significantly longer survival (median OS, 14 months versus 10 months) compared with patients with higher Ki-67 indices. On the other hand, a Ki-67 index of <60% was predictive for response to treatment and survival with temozolomide-based second-line chemotherapy for NEC [8]. Temozolomide was reported to be one of the key drugs in advanced pancreatic NET with a Ki-67 index ranging from 2% to 20% [18, 19]. Considering these facts, NEC with a low Ki-67 index may have clinical or pathological similarity with NET. In this study, amrubicin therapy was effective for NEC with a high Ki-67 index. This suggests that chemotherapy regimens might be administered according to the Ki-67 index or previous chemotherapy regimens in salvage chemotherapy.

Toxicities observed in this study were mainly hematological and similar to previous reports on amrubicin chemotherapy. Although grade 3-4 neutropenia was developed in 40%, the adverse effects were manageable in all cases by careful monitoring of myelosuppression and appropriate dose reduction of amrubicin.

In conclusion, our study showed that amrubicin monotherapy was potentially active and well tolerated for platinum-refractory gastroenteropancreatic NEC.

## Figures and Tables

**Table 1:** Patient characteristics.

Characteristics	*n*
Age	
Median (range)	67 (52–78)
Gender	
Male/female	8/2
PS	
0-1/2	6/4
Primary tumor	
Esophagus	2
Stomach	5
Small intestine	1
Colon	1
Pancreas	1
Stage	
IV/postoperative recurrence	9/1
Metastatic sites	
Lymph node	9
Liver	6
Peritoneum	1
Number of metastatic sites	
1/≥2	2/8
Number of courses of previous chemotherapy	
1/≥2	5/5
Regimens of first-line therapy	
Cisplatin + irinotecan	7
Carboplatin + etoposide	1
Cisplatin + etoposide	2
Ki-67 index (before first-line treatment)	
<60%	4
60%–100%	6

**Table 2:** Toxicity.

	Grade				
	1	2	3	4	3 + 4 (%)
Hematologic					
Leukopenia	3	3	2	0	2 (20)
Neutropenia	4	0	2	2	4 (40)
Anemia	3	2	5	0	5 (50)
Thrombocytopenia	4	0	0	0	0
Total bilirubin elevation	3	0	0	0	0
ALT elevation	7	0	0	0	0
AST elevation	5	0	0	0	0
Hyponatremia	7	2	0	0	0
Hyperkalemia	3	0	0	0	0
Nonhematologic					
Anorexia	6	3	1	0	1 (10)
Nausea	5	3	1	0	1 (10)
Vomiting	1	0	0	0	0
Fatigue	4	3	1	0	1 (10)
Mucositis	3	0	0	0	0
Diarrhea	0	0	0	0	0
Constipation	4	4	0	0	0
